# Modelling cascading failures in networks with the harmonic closeness

**DOI:** 10.1371/journal.pone.0243801

**Published:** 2021-01-25

**Authors:** Yucheng Hao, Limin Jia, Yanhui Wang, Zhichao He

**Affiliations:** 1 State Key Laboratory of Rail Traffic Control and Safety, Beijing Jiaotong University, Beijing, China; 2 School of Traffic and Transportation, Beijing Jiaotong University, Beijing, China; 3 Beijing Research Center of Urban Traffic Information Sensing and Service Technology, Beijing Jiaotong University, Beijing, China; Universite Lumiere Lyon 2, FRANCE

## Abstract

Many studies on cascading failures adopt the degree or the betweenness of a node to define its load. From a novel perspective, we propose an approach to obtain initial loads considering the harmonic closeness and the impact of neighboring nodes. Based on simulation results for different adjustable parameter *θ*, local parameter *δ* and proportion of attacked nodes *f*, it is found that in scale-free networks (SF networks), small-world networks (SW networks) and Erdos-Renyi networks (ER networks), there exists a negative correlation between optimal *θ* and *δ*. By the removal of the low load node, cascading failures are more likely to occur in some cases. In addition, we find a valuable result that our method yields better performance compared with other methods in SF networks with an arbitrary *f*, SW and ER networks with large *f*. Moreover, the method concerning the harmonic closeness makes these three model networks more robust for different average degrees. Finally, we perform the simulations on twenty real networks, whose results verify that our method is also effective to distribute the initial load in different real networks.

## Introduction

Enhancing the ability of real-world networks to resist cascading failures is a hot topic and many scholars have paid a lot of attention to it. In reality, the resilience of infrastructure networks is often affected by random failures or intentional attacks, for example, the large-scale blackout in America, the paralysis of the railway network and the power grid in China due to natural disasters, and so on. To this end, there are many works focusing on infrastructure systems [1–8], communication networks [9–11], and supply networks [12, 13]. Because the load on a node over the capacity causes the failure propagation, how to allocate initial loads is closely related to the robustness of networks.

As a basic measure, the node degree is crucial to cascading models. Initial loads of nodes were dependent on their degrees [14, 15], and the corresponding results showed that the network robustness was improved greatly in the case of a specific value of a parameter. In the same way, initial loads on edges were computed by the node degree [16–19]. Taking into account the degree and the local information, Wang et al. [20] put forward a definition concerning the initial load for the investigation of the network robustness. Furthermore, there is a key finding that the betweenness of a node has a close relationship with the degrees of its adjacent nodes [21, 22]. Consequently, the approach combining the node degree and the degrees of adjacent nodes was presented to obtain loads of nodes [12, 23–25].

Motter et al. [26, 27] found a heterogeneous distribution of loads that depended on the total number of shortest paths in real networks, and explored the failure propagation. Similarly, Mirzasoleiman et al. [28] developed an approach to calculate the weights of edges whose initial loads were calculated by multiplying the betweenness of nodes, and demonstrated that the method with respect to the betweenness makes networks more robust compared with the one with respect to the degree. Since Kim et al. [29] found that a linear relationship between loads and capacities of nodes is relatively rare, the betweenness was used to measure the property of complex systems [30]. Besides, to consider the comprehensive information on a node, Liu et al. [31] studied the computation of initial loads by integrating the node degree and the betweenness.

On the basis of the percolation theory, Buldyrev et al. [32] introduced the framework of the failure propagation in the coupled network. In addition, in the case that initial loads of nodes were determined by the degree [33, 34] and the betweenness [35–39], the robustness of interdependent networks against the cascading failure was discussed. Hong et al. [40] studied the restoration strategy of interdependent networks in which the load on each node was assigned a random value.

Over the past decade, there have been a great number of works concerning the model of cascading failures, but they exist the obvious limitation to calculate initial loads regardless of single networks and interdependent networks. Although we can calculate the node degree easily, the impact of the failure may propagate the other nodes except for neighboring nodes, indicating that calculating loads by the node degree could not be reasonable. In terms of other measures for a node, the importance of a node in the whole network is reflected by its betweenness, but networks with the betweenness are more vulnerable in some cases, which is verified by the detail on the simulations of SW and ER networks in later sections. Besides, for the node with the low degree, its betweenness is likely to equal zero, implying that obtaining the load of this node by its betweenness makes no sense. In order to overcome the defects of previous works, a new definition of the initial loads of nodes is put forward by means of the harmonic closeness and the knowledge of adjacent nodes. Compared with the work [41], by taking into account different measures for a node (including the harmonic closeness, the degree, the betweenness, the random-walk betweenness, the PageRank, and the closeness), we give six methods of calculating the initial load, and make a systematic comparison among them. Scale-free, small-world and random networks with different average degrees are utilized to analyze the advantage of our method. Additionally, in order to discuss the application of this method, we choose twenty real-world networks from different categories to perform case studies. According to the simulation results, it is found that our method can significantly strengthen the robustness of model networks with different average degrees and real-world networks.

## Model

Recently, works [42, 43] investigate the cascading models where the loads on nodes and edges are decided by the harmonic closeness which is defined as follows,

$$hc_{i}=\frac{1}{N-1}\sum_{i\neq j}\frac{1}{d_{ij}}$$ (1)

where *hc*_*i*_ represents the harmonic closeness of node *i*. *N* is the number of nodes and *d*_*ij*_ represents the shortest distance between node *i* and node *j*.

To measure the interaction between a node and its neighboring node, an approach considering the harmonic closeness of adjacent nodes (HA) is present here. Thus, initial load *L*_*i*_(0) on node *i* is given as follows,

$$L_{i}(0)=\delta hc_{i}^{\theta }+(1-\delta )\frac{\sum_{j\in \Gamma_{i}}hc_{j}^{\theta }}{k_{i}}$$ (2)

where *θ*(*θ*>0) represents an adjustable parameter to govern the distribution of loads by the measure of the node while *δ*(0≤*δ*≤1) represents a local parameter that controls the mean interaction of adjacent nodes on a node. *k*_*i*_ stands for the degree of node *i*. Γ_*i*_ stands for the set of nodes that connect with node *i*.

1. Based on the existing model, the capacity *C*_*i*_ of node *i* is given as follows,

$$C_{i}=TL_{i}(0)$$ (3)

where *T*(*T*>1) represents a tolerance parameter. In general, it is difficult to increase *T* to a large value in consideration of the cost. Consequently, the impact of cascading failures can be assessed by the critical threshold *T*_*c*_. Namely, when we remove any node from the set of attacked nodes, the other node keeps its function in the range of *T*≥*T*_*c*_. If *T*<*T*_*c*_, cascading failures will happen.

To reduce the damage of the failure propagation, the node with the high initial load should undertake the more additional load for retaining the normal function. Accordingly, the additional load Δ*L*_*ij*_(*t*) which adjacent node *j* receives from the failed node *i* at step *t* is proportional to its initial load *L*_*j*_,

$$\Delta L_{ij}(t)=L_{i}(t)\frac{L_{j}(0)}{\sum_{k\in \Gamma_{i}}L_{k}(0)}$$ (4)

In the light of the additional load, the load on the adjacent node *j* is updated as follows,

$$L_{j}(t+1)=L_{j}(t)+\Delta L_{ij}(t)$$ (5)

When a load on a node exceeds its capacity, this node malfunctions and causes the cascading failure. Until the load of every node is not larger than its capacity, the cascading failure stops.

## Simulations

In this research, we choose three kinds of model networks, i.e., scale-free networks (SF networks) [44], small-world networks (SW networks) [45], and Erdos-Renyi random networks (ER networks) [46]. SF, SW and ER model networks with *N* = 10000 are respectively constructed for a given proportion *f* of nodes to be removed, and data points are the average results in 20 independent networks. In addition, two attack modes are adopted, i.e., attacks on the node in the descending order of their loads (DL) and attacks on the node in the ascending order of their loads (AL), and then the set of attacked nodes is obtained by the first *N*×*f* nodes. When the loads among nodes are equal to each other, they are randomly attacked.

**Fig 1:**
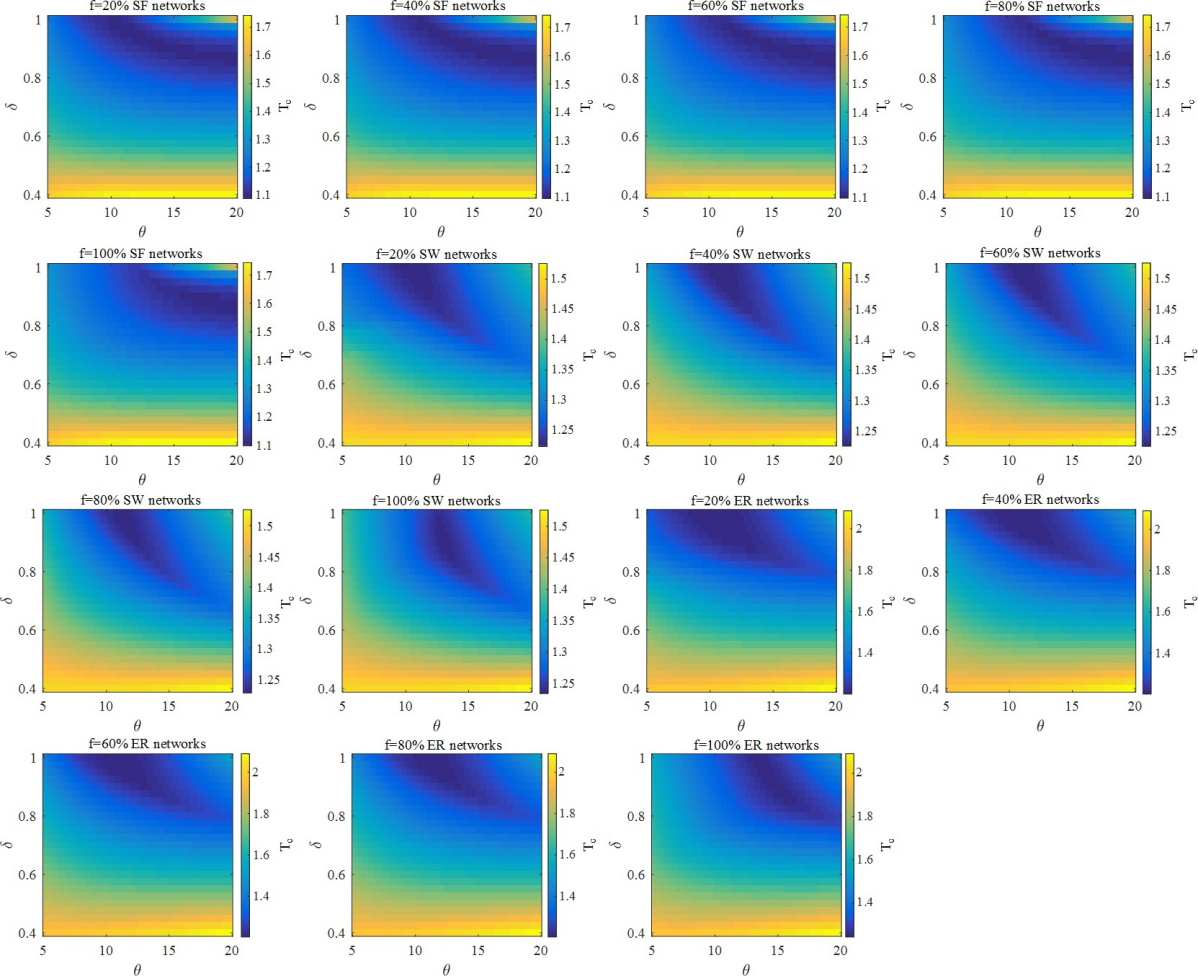
The simulation results of the robustness of SF, SW and ER networks at different *θ*, *δ* and *f* under DL when <*k*> = 4.

To discuss the relationship between parameters and the robustness in networks under DL, we carry out the simulations with different parameters when <*k*> = 4. In Fig 1, we can see the distribution of the robustness in SF, SW and ER networks. These simulations report a striking finding that the optimal range of *δ* is between 0.7 and 1. It implies that in three networks, the local information regarding the adjacent nodes has a marked effect on the optimal distribution of loads. Besides, the range of optimal *δ* remains almost unchanged in SF, SW and ER networks when *f* increases from 20% to 100%. As a result, in terms of the assign of the initial load by HA, the boundary of optimal *δ* has no correlation with the change of *f*. On the contrary, in Fig 1, we can observe that the value of optimal *θ* in these three networks increases with the increase of *f*.

It should be noted that in SF, SW and ER networks no matter what *f* is, optimal *θ* has a strong negative correlation with optimal *δ*. The major reason is that the increased value of *θ* or *δ* is prone to bring about an obvious difference among the initial loads. However, as one of them increases, the failure of the high load node is likely to trigger cascading failures. Thus, for remaining the reasonable difference of the initial loads, the larger optimal *θ*, the smaller optimal *δ*, and vice versa.

**Fig 2:**
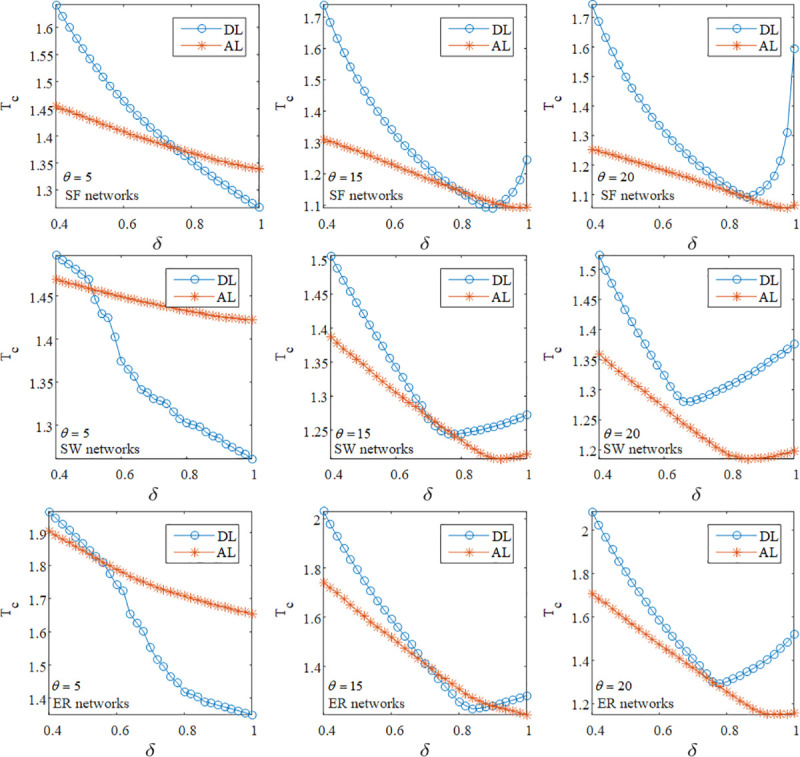
The simulation results of the robustness of SF, SW and ER networks at different *θ* and *δ* under DL and AL when *f* = 10% and <*k*> = 4.

In this part, we discuss the impact of DL and AL on cascading failures for different *θ* and *δ* when <*k*> = 4. Fig 2 illustrates that for different *θ*, the observation of DL and AL could be divided into three categories, and it is more complex than existing works [4, 18, 24]. In the first case, in SF, SW and ER networks with small *θ*, it can be seen a crossing point between the curves of DL and AL. That is to say, the value of *T*_*C*_ under AL is larger than the one under DL when the value of *δ* is large. This counterintuitive phenomenon shows that attacks on the low load node are prone to induce the failure propagation in the case of small *θ* and large *δ*, and this is in agreement with the previous works [4, 24]. In the second case, it can be found an interesting result that when *δ* increases, there are two crossing points between curves of DL and AL in SF, SW, and ER networks at *θ* = 15. This indicates that in the range of too small or too large *δ*, DL can more seriously impair the network robustness compared with AL. In terms of SF, SW and ER networks at *θ* = 20, i.e., the last case, the value of *T*_*C*_ under DL is larger than the one under AL no matter what the value of *δ* is. In this case, the cascading failure may be triggered by the failure of the high load node instead of the one of the low load node.

Additionally, in Fig 2, we can observe that almost all of the curves of AL show a downward trend as *δ* increases in SF, SW and ER networks at different *θ*. This is because the low load node tends to cause failures of its adjacent nodes when the difference of loads is not significant. Moreover, the larger the value of *δ*, the greater the difference among the initial loads. Therefore, attacks on the low load node have less impact on the network by increasing the value of *δ*.

**Fig 3:**
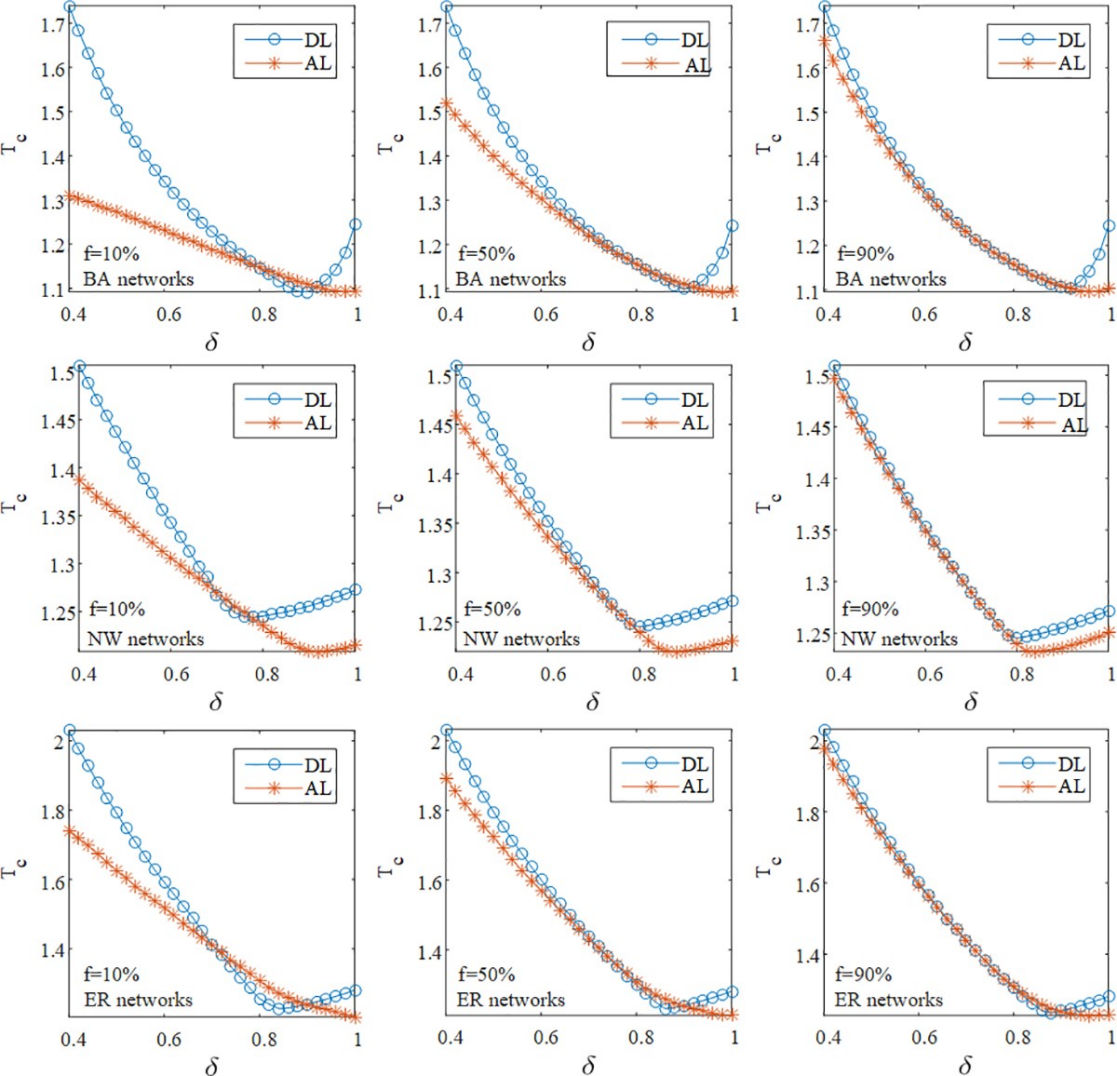
The simulation results of the robustness of SF, SW and ER networks at different *f* and *δ* under DL and AL when *θ* = 10 and <*k*> = 4.

In order to analyze the relationship between attack modes and the number of attacked nodes, we perform the comparison of DL and AL in the above model networks at different *f* and *δ* when <*k*> = 4. In Fig 3, it is evident that at *f* = 10% or *f* = 50%, the value of *T*_*C*_ under AL is larger than the one under DL in these model networks when *δ* is approximately equal to 0.8. It means that it is easy to induce the failure propagation owing to the removal of the low load node in networks with *δ* = 0.8 when the number of attacked nodes is not too high. Furthermore, nodes that lead to cascading failures under DL are basically the same as those under AL when *f* is large, therefore increasing the value of *f* makes curves of DL and AL more similar.

In the field of complex networks, many characteristics of the node have been studied widely, such as the node degree, the node betweenness, the random-walk betweenness, the PageRank, the closeness and so on. It is well-known that the degree can reflect the local information on a node while the harmonic closeness, the betweenness, the PageRank, and the closeness can reflect the global information. In order to carry out the systematic comparison of methods of calculating loads by different measures for the node, the approaches of computing the initial load based on the degree of the adjacent node (DA), the betweenness of the adjacent node (BA), the random-walk betweenness of the adjacent node (RBA), the PageRank of the adjacent node (PA), and the closeness of the adjacent node (CA) are respectively given

$$W_{i}=\delta k_{i}^{\theta }+(1-\delta )\frac{\sum_{j\in \Gamma_{i}}k_{j}^{\theta }}{k_{i}}$$ (6)

$$W_{i}=\delta b_{i}^{\theta }+(1-\delta )\frac{\sum_{j\in \Gamma_{i}}b_{j}^{\theta }}{k_{i}}$$ (7)

$$W_{i}=\delta rb_{i}^{\theta }+(1-\delta )\frac{\sum_{j\in \Gamma_{i}}rb_{j}^{\theta }}{k_{i}}$$ (8)

$$W_{i}=\delta p_{i}^{\theta }+(1-\delta )\frac{\sum_{j\in \Gamma_{i}}p_{j}^{\theta }}{k_{i}}$$ (9)

$$W_{i}=\delta c_{i}^{\theta }+(1-\delta )\frac{\sum_{j\in \Gamma_{i}}c_{j}^{\theta }}{k_{i}}$$ (10)

where *b*_*i*_, *rb*_*i*_, *p*_*i*_ and *c*_*i*_ represent the values of the betweenness, the random-walk betweenness, the PageRank, and the closeness of node *i*, respectively.

When *θ* and *δ* are equal to specific values respectively, *T*_*C*_ is minimized for a given *f*, called optimal *T*_*C*_. In this section, we investigate whether optimal *T*_*C*_ obtained by AH is lower than the ones obtained by the above methods of calculating loads.

**Fig 4:**
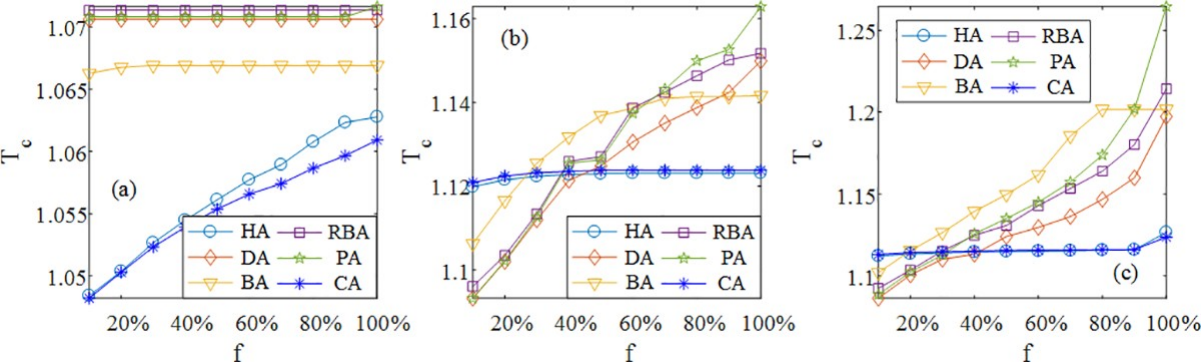
The simulation results of the robustness of SF (a), SW (b) and ER (c) networks at different *f* under DL when <*k*> = 8 based on HA, DA, BA, RBA, PA and CA with optimal *θ* and *δ*.

Fig 4(A) shows that in SF networks, there exists little change in every curve except for HA and CA. Due to the similarity of the definition between HA and CA, their curves hardly differ from each other. As *f* increases, the curves of HA and CA steadily rise, but at different *f*, the values of optimal *T*_*C*_ calculated by HA and CA are significantly lower than others. Furthermore, the smaller *f* is, the more obvious the advantage of HA and CA in SF networks is. The above results mean that scale-free networks with HA and CA can more effectively resist cascading failures regardless of *f* compared with the ones with other methods, which is more evident especially for the case of less attacked nodes. In contrast, the SF network with RBA is the most fragile to cascading failures.

In Fig 4(B) and 4(C), it can be found that in SW and ER networks, the curves of HA and CA are slightly higher than other curves in the range of small *f*. However, the values of optimal *T*_*C*_ obtained by HA and CA almost keep constant, while the ones obtained by other methods greatly increase with the increase of *f*. In particular, when *f* increases to 50%, the curves of HA and CA are lower than those of other approaches in SW and ER networks. The simulation demonstrates that HA and CA are the more efficient methods to distribute loads when many nodes are attacked in homogeneous networks. In addition, the curves of BA and PA for SW and ER networks are higher, on the whole, indicating that although the measures concerning the betweenness and the PageRank can represent the importance of a node on the entire network, the methods with those two measures tend to result in the high vulnerability compared with other methods.

Besides, we also focus on the special case, i.e., *f* = 100%, which means that attacking any node cannot induce cascading failures. As *f* changes from 0% to 100%, the curves of different methods of calculating loads can be divided into two categories: gradual curves (e.g., the case of HA in ER networks) and steep curves (e.g., the case of HA in SF networks). We adopt DL to attack the high load nodes, hence if the curve is virtually unchanged when *f* increases, the node with the high load will decide whether cascading failures are caused in the case of *f* = 100%. On the contrary, the steep curve means that optimal *T*_*C*_ at *f* = 100% depends on the node with the low load instead of the ones with the high load. Thus, in this case, if the low load node does not cause the failure propagation, no cascading failures will occur. In Fig 4, it is clear that for most of curves, the value of optimal *T*_*C*_ is not sensitive to the change of *f* in SF networks, which is contrary to the cases of SW and ER networks. As a consequence, it could be concluded that the low (high) load node plays a key role in the occurrence of cascading failures in SF (SW and ER) networks for most methods of calculating loads in consideration of the attack on any node.

**Fig 5:**
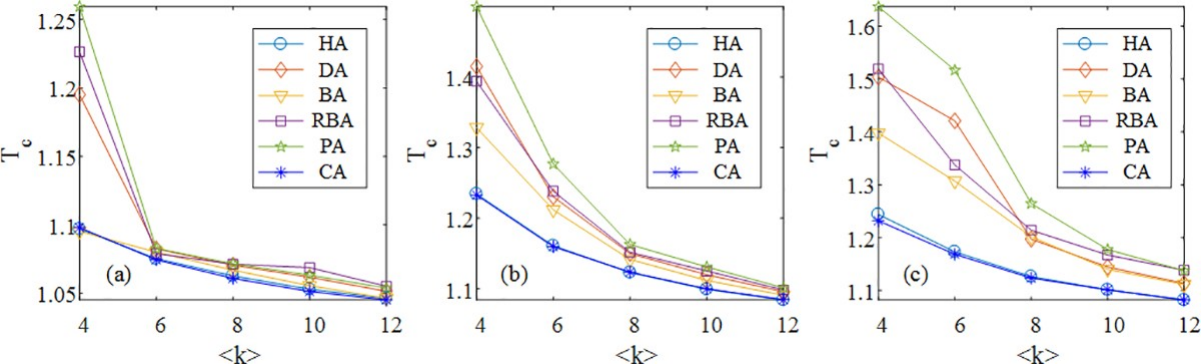
The simulation results of the robustness of SF (a), SW (b) and ER (c) networks at different <*k*> under DL when *f* = 100% based on HA, DA, BA, RBA, PA and CA with optimal *θ* and *δ*.

Generally speaking, the average degree <*k*> is strongly related to the network robustness, therefore considering SF, SW and ER networks at different <*k*>, we conduct the simulation experiments based on these six methods. In Fig 5, there is an important finding that the values of optimal *T*_*C*_ calculated by HA and CA are lower than others for different <*k*> no matter what the kind of the network is. In particular, for small <*k*>, the advantage of HA and CA is more significant in model networks. These results show that it is effective to adopt the harmonic closeness and the closeness for the computation of initial loads regardless of the number of edges in networks. Furthermore, with the increase of <*k*>, the difference of curves becomes slighter. Additionally, in three networks mentioned above, we find a common ground that the curve of PA is high for the small value of <*k*>.

**Fig 6:**
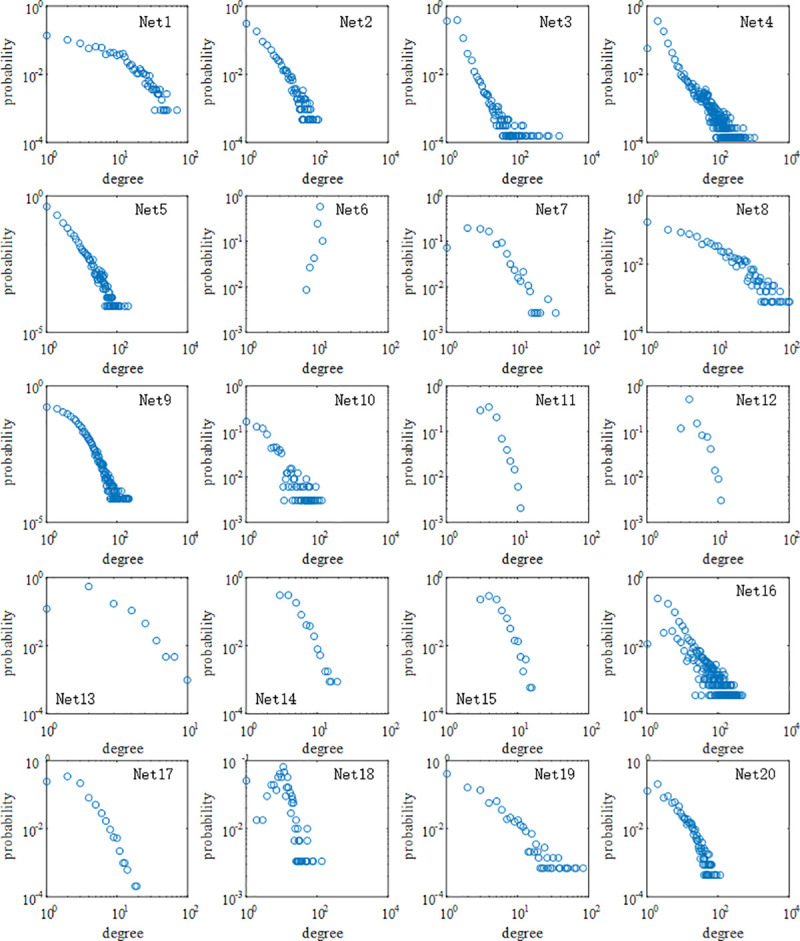
Distribution of the node degree in real-world networks.

Model networks are helpful for understanding the behavior of real systems, but they hardly reflect the feature of real networks because of the ideal conditions. To fully discuss the dynamics of cascading failures in real systems, we apply methods mentioned above to different kinds of real networks. Note that our aim is to analyze the failure propagation in the giant component of undirected networks without self-loops and parallel edges, therefore, in these real networks, nodes out of the giant component, self-loops and parallel edges are deleted. The data of real networks is listed in Table 1 and the distribution of the node degree is shown in Fig 6.

**Table 1:** Basic data about real networks.

Category	Network	Network ID	Nodes	Edges
Technological Network	Email [47]	Net1	1133	5451
	Router network [48]	Net2	2113	6632
	Autonomous system of Internet [49]	Net3	6474	12572
	WHOIS network [50]	Net4	7476	56943
	Network of users of the Pretty-Good-Privacy algorithm for secure information interchange [51]	Net5	10680	24316
Social Network	Network of American football games [52]	Net6	115	613
	Coauthorship in network science [53]	Net7	379	914
	Interactions between science in society actors on the Web [54]	Net8	1272	6454
	Facebook page networks [55]	Net9	14113	52126
Infrastructure network	US airlines [56]	Net10	332	2126
	Power grid with 494 nodes [57]	Net11	494	586
	Power grid with 662 nodes [58]	Net12	662	906
	European road network [59]	Net13	1039	1305
	Power grid with 1138 nodes [60]	Net14	1138	1458
	Power grid with 1723 nodes [61]	Net15	1723	2394
	Network of flights [62]	Net16	2905	15645
	US power grid [63]	Net17	4941	6594
Biological Network	Elegans neural network [64]	Net18	297	2148
	Network of disorders and disease genes [65]	Net19	1419	2738
	Protein-protein interaction network in yeast [66]	Net20	2224	6609

In Fig 6, it is apparent that there are a few nodes with the high degree while most of nodes have the low degree, and there exists a fat-tailed distribution in Nets 1–5, 8–10, 16, 19 and 20. Consequently, the above real networks possess the scale-free property and belong to the heterogeneous network. On the contrary, other real networks belong to the homogeneous network.

**Fig 7:**
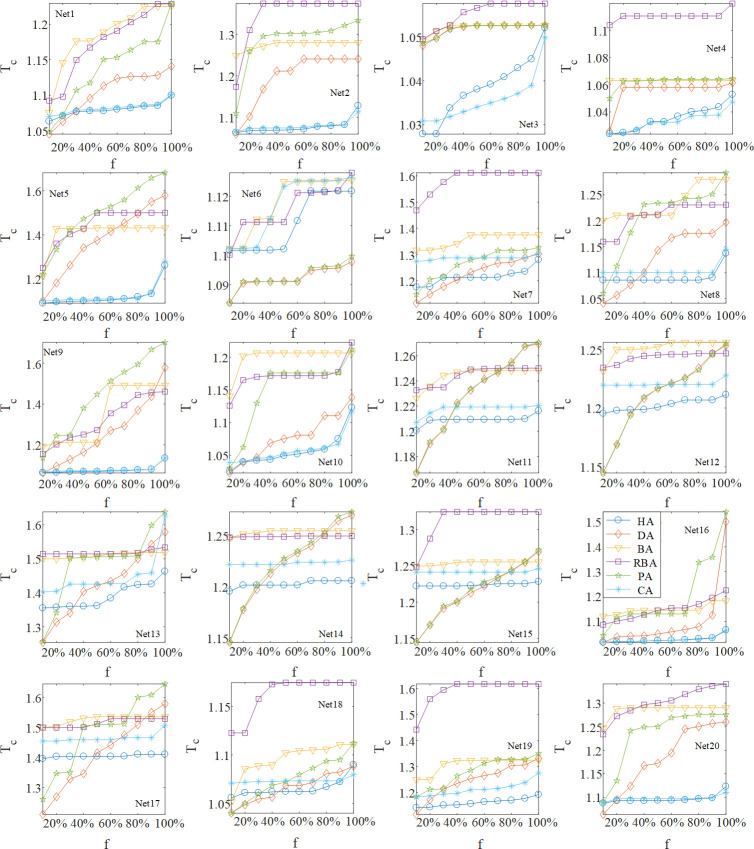
Comparison of HA, DA, BA, RBA, PA, and CA with optimal *θ* and *δ* in real-world networks at different *f*.

Here we carry out the simulation experiment on real networks with different methods to calculate loads. From Fig 7, it can be found that Nets 1–5, 8–10, 16, 19 and 20 with HA have small *T*_*c*_ in most cases, hence our method outperforms other methods in real networks with the scale-free property. Especially for Nets 2, 4, 5, and 9 with HA, the values of optimal *T*_*c*_ are significantly smaller than others for an arbitrary value of *f*. As a result of the scale-free property in these real-world networks, the observation of HA in the networks is similar to that in SF networks. In terms of Nets 7, 11–15, and 17–18, the curves of HA are insensitive to the number of attacked nodes. When *f* increases to a large value, the values of optimal *T*_*c*_ obtained by HA are the smallest in corresponding networks, indicating that HA can make these real networks more resilient to the failure propagation when a large number of nodes are attacked. In addition, according to the simulation results, there is no significant difference between the change of curves in SW, ER networks and that in the above real networks, which also proves that these real networks belong to the homogeneous network. Note that in model networks, the result of HA is similar to the one of CA, while we can see that Nets 3, 7, 8, 11–15, 17 and 19 possess the stronger robustness by using HA. This means that HA has wide applicability for distributing the load in real-world networks. To sum up, HA is able to greatly enhance the robustness of real networks with different topology structures.

## Discussion and conclusions

For most of the studies on cascading failures, initial loads on nodes are determined by their degree or betweenness. In this paper, we propose an approach to obtain initial loads by means of the harmonic closeness and the knowledge of adjacent nodes. In order to control the strength of the initial load on a node, *θ* reflecting the property of the node and *δ* reflecting the impact of adjacent nodes are adopted. According to the simulations on model networks (SF, SW and ER networks) and real networks, we investigate the impact of the parameters on *T*_*C*_ under different *f*, finding that there is a negative relationship between optimal *θ* and *δ* for a certain value of *f*. Through the analysis of different attack modes, it is revealed that the failure of the low load node is more likely to trigger the cascading failure in some cases. Furthermore, we obtain a key result that the method with the harmonic closeness to calculate loads reduces the probability of triggering the failure propagation in SF networks and real networks with the scale-free property compared with other methods regardless of *f*. For SW, ER, and real networks with the homogeneous degree distribution, our method strengthens their robustness in the case of large *f*. In addition, this method is also effective to calculate the initial load in SF, SW and ER networks at different <*k*> when *f* = 1. The above findings indicate that it is reasonable to allocate initial loads based on the harmonic closeness for preventing cascading failures. We believe that this work should be in favor of the design of real-world systems and the theoretical analysis of cascading failures.
